# A novel community-based study to address disparities in hypertension and colorectal cancer: a study protocol for a randomized control trial

**DOI:** 10.1186/1745-6215-14-287

**Published:** 2013-09-08

**Authors:** Joseph Ravenell, Hayley Thompson, Helen Cole, Jordan Plumhoff, Gia Cobb, Lola Afolabi, Carla Boutin-Foster, Martin Wells, Marian Scott, Gbenga Ogedegbe

**Affiliations:** 1 Center for Healthful Behavior Change, New York University School of Medicine, 227 E. 30th St., 6th Floor, Room 637, New York, NY 10016, USA; 2 Population Studies and Disparities Research Program, Karmanos Cancer Institute Department of Oncology, Wayne State University School of Medicine, 4100 John R - MM03CB, Detroit, MI 48201, USA; 3 Center for Healthful Behavior Change, New York University School of Medicine, 227 E. 30th St., 6th Floor, Room 632C, New York, NY 10016, USA; 4 Center for Healthful Behavior Change, New York University School of Medicine, 227 E. 30th St., 6th Floor, Room 632B, New York, NY 10016, USA; 5 Center for Healthful Behavior Change, New York University School of Medicine, 227 E. 30th St., 6th Floor, Room 632A, New York, NY 10016, USA; 6 Formerly with the Center for Healthful Behavior Change, 900 Lenox Rd, Apt 2B, Brooklyn, NY 11203, USA; 7 505 East 70th Street, Helmsley Tower, 4th Floor, New York, NY 10021, USA; 8 301 Malott Hall, Cornell University, Ithaca, NY 14853, USA; 9 St. Luke’s and Roosevelt Hospitals, 555 W. 57th Street, Suite 5-43, New York, NY 10019, USA; 10 Center for Healthful Behavior Change, New York University School of Medicine, 227 E. 30th St., 6th Floor, Room 633, New York, NY 10016, USA

**Keywords:** Hypertension, Patient navigation, African Americans, Barbershops

## Abstract

**Background:**

Black men have the greatest burden of premature death and disability from hypertension (HTN) in the United States, and the highest incidence and mortality from colorectal cancer (CRC). While several clinical trials have reported beneficial effects of lifestyle changes on blood pressure (BP) reduction, and improved CRC screening with patient navigation (PN), the effectiveness of these approaches in community-based settings remains understudied, particularly among Black men.

**Methods/design:**

MISTER B is a two-parallel-arm randomized controlled trial that will compare the effect of a motivational interviewing tailored lifestyle intervention (MINT) versus a culturally targeted PN intervention on improvement of BP and CRC screening among black men aged ≥50 with uncontrolled HTN who are eligible for CRC screening. Approximately 480 self-identified black men will be randomly assigned to one of the two study conditions. This innovative research design allows each intervention to serve as the control for the other. Specifically, the MINT arm is the control condition for the PN arm, and vice-versa. This novel, simultaneous testing of two community-based interventions in a randomized fashion is an economical and yet rigorous strategy that also enhances the acceptability of the project. Participants will be recruited during scheduled screening events at barbershops in New York City. Trained research assistants will conduct the lifestyle intervention, while trained community health workers will deliver the PN intervention. The primary outcomes will be 1) within-patient change in systolic and diastolic BP from baseline to six months and 2) CRC screening rates at six months.

**Discussion:**

This innovative study will provide a unique opportunity to test two interventions for two health disparities simultaneously in community-based settings. Our study is one of the first to test culturally targeted patient navigation for CRC screening among black men in barbershops. Thus, our study has the potential to improve the reach of hypertension control and cancer prevention efforts within a high-risk population that is under-represented in primary care settings.

**Trial registration:**

ClinicalTrials.gov, NCT01092078

## Background

Black men have the greatest burden of premature death and disability from hypertension (HTN) in the United States [1]. A major reason for the disproportionate morbidity and mortality from HTN and its complications in black men is poor blood pressure (BP) control [2]. Improving BP control leads to significant cardiovascular risk reduction and can be achieved through interventions targeted at therapeutic lifestyle changes (TLC) [3-11]. Several clinical trials have reported beneficial effects of lifestyle changes, such as increased fruit and vegetable intake, decreased sodium intake, increased physical activity, increased medication adherence and smoking cessation on BP reduction [3,7,10,12-14]. As a result, lifestyle changes have been adopted by various HTN treatment guidelines as a standard first-line or adjunctive therapeutic option [15]. Despite the proven efficacy of lifestyle changes in lowering BP in academic research settings [3-8,12,13], there is limited data on the effectiveness of TLC interventions in community-based settings in this high-risk population. Of hypertensive adults in the United States, 43% are black compared to 33.9% white [16]. Black men in particular have experienced higher mortality rates resulting from hypertension when compared to white men. In 2008, the death rate among black men from hypertension was 50.3 per 100,000 compared to 16.5 per 100,000 for white men [16]. Increased rates of cardiovascular disease have contributed to disparities in life expectancy, where life expectancy for whites exceeded that for blacks by 5.3 years in 2003, with the lowest life expectancy experienced by black men (69 years) [1]. While the disproportionately high rate of hypertension-related morbidity and mortality is often addressed in the literature, the epidemic of colorectal cancer (CRC) among black men is comparatively under-appreciated. Black men have the highest incidence of CRC (67.2 per 100,000 compared to 56.1 per 100,000 among white men) and the highest CRC mortality (30.5 per 100,000 compared to 20.6 per 100,000 among white men) in the US [17,18], yet have significantly lower screening rates than whites nationally [19].

One explanation for the disproportionate mortality due to CRC may be that black men are less likely than white men to be diagnosed at an early stage of the disease, leading to decreased survival rates [20]. Lower rates of early stage diagnosis may be due in part to lack of timely screening, since CRC screening leads to identification and often, curative excision of precancerous polyps and early cancers. Current national guidelines recommend that all adults of average CRC risk be screened starting at age 50, with the frequency of screening varying from 1 to 10 years based on the type of screening test used and personal and familial history. However, black men are less likely to be screened before the age of 65 than white men. Even in New York City, where disparities in overall CRC screening have been reduced over the past decade, racial differences in age at screening, early stage diagnosis and CRC mortality persist. Thus, for black men, a focus on early CRC screening is particularly important.

Several approaches have been shown to be successful in increasing CRC screening rates [21-23]. One such intervention is patient navigation, defined as “assistance offered to patients, survivors, families, and caregivers to help them access and chart a course through the healthcare system” and overcome barriers to healthcare [24]. Patient navigation has demonstrated efficacy in increasing CRC screening rates when delivered in practice-based settings [24-32], particularly among minority groups. However, as with TLC for HTN control, the effectiveness of patient navigation programs in improving the CRC screening rates in black men has not been tested in non-clinical, community-based settings. For both hypertension and CRC, the translation of evidence-based approaches into community-based settings is necessary for reducing the noted racial disparities in hypertension-related outcomes and CRC mortality in black men. Barbershop-based HTN outreach programs are becoming common nationwide, but whether they are an effective approach for improving HTN control among black men is unknown due to a dearth of evaluation research. Furthermore, no known CRC screening interventions have been tested in the barbershop setting.

Black-owned barbershops are rapidly gaining traction as potential community partners for health promotion programs targeting HTN as well as diabetes, prostate cancer and other diseases that disproportionately affect black men [33-37]. Barbershops hold special appeal for community-based intervention trials, as they are a cultural institution that draws a large and loyal male clientele and provides an open forum for discussion of numerous topics, including health, with influential peers. A focus on community-based settings is particularly important among black men, a group that is less likely to access primary care health care settings [38]. The one randomized barbershop-based hypertension trial in the peer-reviewed literature (the BARBER-1 trial), was promising, demonstrating that a program of continuous BP monitoring and peer-based health messaging in a barbershop can: (1) be implemented by lay health workers rather than research personnel [39], and (2) improve BP control in the barbershop compared to printed educational materials [38]. Building on these promising results, we propose that, given its historical significance as a trusted community setting [33], the barbershop is not only a valuable delivery channel for evidence-based interventions for hypertension, but may be a useful setting to address CRC prevention as well. In this article, we describe an innovative randomized controlled trial that simultaneously evaluates the effectiveness of two evidence-based interventions set in black barbershops targeted at BP reduction and CRC screening in black men. Utilizing a cross-randomized design, we eliminate the need for a true control group thereby increasing community trust for the study, given that no participant would be left out of receiving an intervention.

The current study, the *M*ulti-Intervention Study to *I*mprove CRC *S*creening and *t*o *E*nhance *R*isk Reduction in *B*lack Men (MISTER B), advances health services research in two ways: 1) by translating clinically-proven interventions for two ameliorable disease conditions that disproportionately impact a high risk population to a culturally-important community-based setting, and 2) by testing two community-based interventions for two different high-impact conditions simultaneously in a randomized controlled fashion.

### Study aims

The primary aim of this study is to evaluate the effect of two interventions on BP reduction and CRC screening among 480 self-identified black men aged ≥50 years with uncontrolled HTN and in need of CRC screening: 1) a lifestyle intervention delivered through telephone-based motivational interviewing (MINT) to reduce blood pressure; and 2) a culturally-tailored patient navigation (PN) intervention delivered by community health workers to promote CRC screening. We hypothesize that among enrolled participants, 1) those randomized to the lifestyle MINT intervention will have lower BP compared to those randomized to the patient navigation intervention at six months, and 2) those randomized to the patient navigation intervention will have higher CRC screening rates compared to those randomized to the lifestyle intervention at six months.

## Methods/design

### Study design

**Figure 1:**
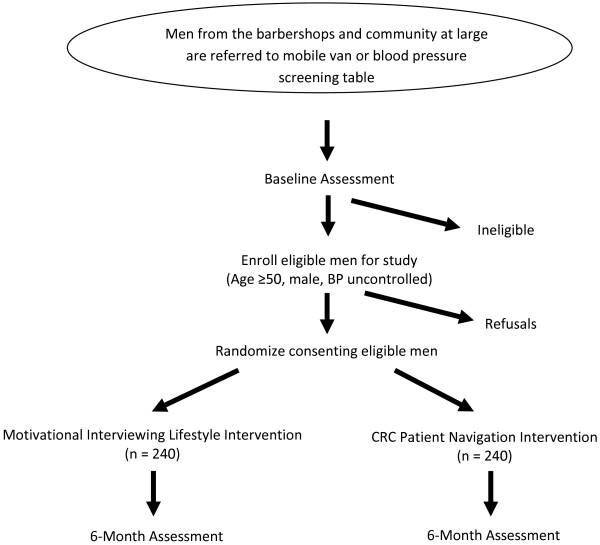
Study design.

MISTER B is a two-parallel-arm randomized controlled trial that will compare the effect of a MINT lifestyle intervention, and a culturally targeted PN intervention on improvement of BP and CRC screening among black men aged >50 with uncontrolled HTN who are eligible for CRC screening (see Figure 1).

#### Advantages of the study design

This innovative research design will allow each intervention to serve as the control for the other. Specifically, the MINT arm will be the control condition for the PN arm, and vice-versa, in that the MINT intervention arm will receive usual counseling for colon cancer screening, and the PN arm will receive usual counseling about appropriate lifestyle changes to improve BP. We believe this novel, simultaneous testing of two community-based interventions in a randomized fashion is an economical and yet rigorous strategy that enhances the acceptability of the project to the community because all participating men will receive a potentially beneficial intervention (that is, no one receives a ‘placebo’. See Figure 1). The MINT intervention will be conducted by trained research assistants, while the PN intervention will be delivered by trained community health workers. Assessments are conducted at the baseline (visit 1) and six months follow-up (visit 2).

Though we are testing interventions simultaneously within the same trial, we do not see the interventions as tackling BP and CRC in tandem. The intervention for BP is distinct from the intervention for CRC, even though the interventions are being tested simultaneously. If these interventions prove successful in our study, successful translation of these interventions would not necessitate that the BP and CRC interventions be delivered together, as they are not delivered together in the study. The interventions are not necessarily complementary, but were chosen because they are similarly high-impact public health problems for black men, and are conducive to community-based delivery in a population that is under-represented in primary care clinical settings.

#### Challenges of the study design

Given that randomization will occur at the level of the individual, and the socially interactive nature of the recruitment setting, wherein participants randomized to different arms will presumably talk to each other, there is potential for some between-arm contamination, which may dampen the observable results in either arm. To address this potential threat to the validity of the study design, we have added important safeguards to minimize contamination. First, both the MINT and PN interventions are highly tailored to address participant’s specific concerns and barriers through individualized counseling strategies implemented over multiple sessions. Each intervention includes several interdependent components, reflecting a complexity that significantly reduces each arm’s vulnerability to contamination. These intervention characteristics, along with the complexity of the target behaviors (lifestyle changes for BP control or colonoscopy) make it unlikely that any exposure to activities, participants or interventionists across study arms would have a significant effect on individual outcomes. Second, we have employed an additional safeguard to minimize contamination: MINT interventionists will be trained separately from PN interventionists, and each interventionist will be trained to do MINT only or PN only. There will be no “cross-training”, thereby minimizing “bleed” of one intervention into another.

#### Eligibility criteria

Uncontrolled HTN is defined in accordance with current consensus clinical hypertension guidelines (Seventh Report of the Joint National Committee on Prevention, Detection, Evaluation, and Treatment of High Blood Pressure (JNC-7)) as an average BP that fulfills criteria of systolic blood pressure (SBP) ≥130 or diastolic blood pressure (DBP) ≥80 mmHg for those with diabetes or chronic kidney disease, and SBP ≥135 or DBP ≥85 mmHg for all others [15]. Criteria for CRC screening eligibility (based on multi-society consensus guidelines) include: 1) no colonoscopy in the last 10 years; 2) no flexible sigmoidoscopy, Digital Contrast Barium Enema or CT-colonoscopy in the last 5 years, or 3) no Fecal Immunochemical Test (FIT) or Fecal Occult Blood Test (FOBT) in the last 12 months. In order to be eligible for the study a man must 1) self-identify as black or African American, 2) be age 50 or over, 3) have uncontrolled HTN, 4) be in need of CRC screening, 5) have a working phone, and 6) be fluent in English. Participants will be excluded if they do not meet any of these criteria. Approximately 480 self-identified black men who meet eligibility criteria will be randomly assigned to one of the two study conditions.

#### Ethical approval

The study has been approved by the Institutional Review Board at New York University School of Medicine (protocol #09-0151). The study will be monitored by a Data Safety and Monitoring Board.

### Study sites and population

MISTER-B will be conducted in black-owned barbershops throughout New York City. Black-owned barbershops are a major small business enterprise in urban centers across the United States [33]. In Harlem alone, there are more than 75 barbershops that cater exclusively to black men. The black-owned barbershop is a popular community site where black men of all socioeconomic strata gather frequently and feel comfortable discussing important issues in their lives [33]. Black barbers are influential peers with a long history of shaping public opinion [33-37]. Previous studies indicate that the average customer has had the same barber for over a decade and visits the same barber for a haircut every week or two [39]. Emerging evidence suggests barbershops constitute an existing and unique community setting and peer network that can be utilized to improve cardiovascular health and cancer prevention in the black male community.

#### Study proceduresBarbershop recruitment

**Figure 2:**
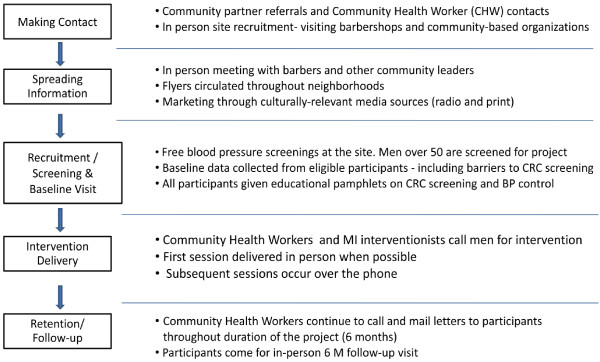
Study procedures.

The recruitment process will begin with the recruitment of barbershops to serve as recruitment sites (see Figure 2). Barbershops will be recruited through word of mouth and by neighborhood tour accompanied by cold recruitment. First, neighborhoods will be identified by examining data from the US Census data and the New York City Department of Health and Mental Hygiene to determine which neighborhoods have large populations of blacks, relatively high incidence of HTN and low rates of timely screening colonoscopy. Staff experience with neighborhoods in New York City and knowledge of traffic patterns in various business districts will also inform where site recruitment is conducted. As more shops are recruited, barbershop referrals from participants, study investigators and barbershop owners will also be approached. For each shop, the study staff will discuss the study with the shop owner or manager and present a written description of study procedures, benefits and expectations. If the shop is interested in participating, the staff and the shop will set up a mutually agreeable time to do an eligibility screening event for the study. Leading up to the eligibility screening, the study staff will work with the owner or manager to advertise in the neighborhood by distributing flyers about the study.

#### Participant recruitment and eligibility screening

Participants will be recruited from barbershops that have agreed to participate in the study. For this purpose, we will park an all-purpose mobile health van at the barbershop storefronts. The van is part of an existing community health promotion program directed by one of the investigators (MS). When the van is not available, we will set up our blood pressure monitors inside the barbershop. Barbers in the participating barbershops will refer potentially eligible customers to the van or to our table. Once a potential participant is referred, the research assistant (RA) will approach him to complete the eligibility screen. As part of the screening, the RA will assess need for CRC screening in each participant, defined as not having 1) a colonoscopy in the last 10 years; 2) flexible sigmoidoscopy, digital contrast barium enema or CT-colonoscopy in the last five years, or 3) FIT or FOBT in the last 12 months. Blood pressure will be measured three times as per the American Heart Association measurement protocol with a validated automatic oscillometric BP monitor, and the average reading will be considered to determine study eligibility. Only those men that meet the eligibility criteria will be invited to participate in the study.

#### Informed consent process

A waiver of signed informed consent was obtained from the IRB for the screening portion of the recruitment process as no identifiable personal health information will be collected for screening purposes. Recruitment staff will acquire verbal informed consent from the participants prior to screening for eligibility. For each eligible participant who decides to enroll in the study, recruitment staff will obtain written informed consent.

#### Data collection

Immediately following the consent process, eligible participants will complete the baseline data collection process which will include a 30- to 45-minute structured interview. The interview will be conducted by trained research assistants, data will be recorded on interview forms, and the interviews will be audio-recorded for fidelity. The baseline interview will consist of structured questions and questionnaires on demographics, psychosocial characteristics, such as self-efficacy and attitudes toward CRC screening comorbidity, and self-reported behaviors associated with the primary and secondary outcomes. See Table 1 for a complete list of study measures. All data will be collected at the barbershop at the time of recruitment. If the participant does not have time to complete all study procedures, an appointment will be made within two weeks of recruitment to complete the remaining procedures. Participants will also be offered the possibility of coming to the study office to complete data collection if it is more convenient for the participant. Follow-up data will be collected six months after each participant is enrolled in the study. Study staff assigned to collect data will make an appointment with each participant by telephone to meet the study staff at the participating barbershop or to meet study staff at the study office to complete the data collection process.

**Table 1:** Study measures

**Measures**	**Baseline**	**6 months**
**Physiological Measures**		
**Blood pressure measurements**	**X**	**X**
**Height, Weight, Waist circumference, and Body Mass Index**	**X**	**X**
**Self-report Measures**		
**Participant demographics**	**X**	**X**
		**(selected questions)**
**Medical Comorbidity (Charlson Comorbidity Index)**	**X**	**X**
**Medication adherence (4-Item Morisky Questionnaire)**	**X**	**X**
**International Physical Activity Questionnaire (IPAQ- Short Version)**	**X**	**X**
**Diet (2-Item Food Frequency Questionnaire)**	**X**	**X**
**Intrinsic Motivation (Treatment Self-regulation Questionnaire) (Diet/Physical Activity version)**	**X**	**X**
**Self-Efficacy (Diet/Physical Activity Self-Efficacy Questionnaire)**	**X**	**X**
**Pros and Cons of Colonoscopy**	**X**	**X**
**Fear of Colonoscopy**	**X**	**X**
**Self-Efficacy Regarding CRC Screening**	**X**	**X**
**Intention and Behavioral Control**	**X**	
**Thompson BQ Healthcare Trust Scale**	**X**	**X**
**Assessment of CRC completion screening**		**X**
**Healthy Lifesyle Changes Follow-up Questionnaire**		**X**
**Decisional Regret**		**X**
**Health Literacy (Newest Vital Sign)**	**X**	
**Intervention Satisfaction**		**X**

#### Randomization

Following the determination of eligibility, consent procedures, and completion of baseline self-report and psychosocial measures, individual-level randomization will occur via a randomization table generated by the study statistician. Participants will be randomized in 1:1 ratio to either the MINT lifestyle intervention or the CRC patient navigation intervention.

Participants will be informed of the results of randomization by an RA at the time of the baseline interview. At this time, the RA will discuss the MINT or the PN intervention/counseling session schedule, and answer any additional questions. Given the nature of the intervention, it is impossible to blind the RAs to the assigned condition. However, the dimension that the RA’s knowledge of the assigned condition could possibly affect is the BP measurement - one of the primary outcomes of this study. To reduce the potential for this bias, we will use an automated BP device (Vital Signs Monitor 300 Series; Welch Allyn; Skaneateles Falls, NY, USA). The other primary outcome, receipt of colon cancer screening, cannot be influenced by the RA’s knowledge of the assigned condition.

#### Participant reimbursement

Participants will receive $15 at the completion of baseline assessment, $20 for completing the majority of the intervention sessions and $20 at the completion of the six-month visit ($55 total) for their time and effort in the study. All participants will also receive free metro cards to help facilitate transportation to and from the study site for the six-month visit. After completing the baseline interview, participants will also receive a voucher for a free haircut to be redeemed at a participating barbershop.

### Description of the interventions

#### MINT lifestyle intervention

Motivational Interviewing (MINT) is an empirically tested, client-centered counseling approach designed to motivate people for change by helping them recognize and resolve the discrepancy between their present behavior and future personal goals and values [40,41]. Several behavioral intervention trials that have included black men (range 12 to 35% of the study population) [42-46], have demonstrated the potential efficacy of MINT in this population. The content of the intervention will be based on established hypertension treatment guidelines, which recommends weight loss (if overweight), limiting sodium and alcohol intake, smoking cessation, medication adherence, regular physical activity and eating a low-fat diet that is rich in fruits and vegetables [15]. As such, all participants will be given the NIH/NHLBI *“Your Guide to Lowering Blood Pressure”* booklet as well as the NIH/NHLBI DASH diet booklet titled *“Facts about the DASH Eating Plan”* and guided through the topics by the research assistant during the baseline session. The methodology, structure and the content of the MINT counseling sessions will be patterned after our recently completed and successful practice-based trial [47].

The MINT sessions will focus on individual needs to tailor intervention strategies to the participant’s personal context, including social support, specific behavior change goals, problem-solving and maintaining motivation during challenging situations (see Table 2). The men randomized to this group will receive four MINT sessions on lifestyle behaviors. The first MINT session will occur in person immediately after baseline data collection at the barbershop and the subsequent three sessions will occur over the phone. The frequency will be monthly for the first two months and then bi-monthly for the next four months, with each session lasting up to 30 minutes. After the introductory session, each subsequent session will include the following steps: 1) assessing the participant’s motivation and confidence in engaging in a given behavior - physical activity, taking medication as prescribed, smoking cessation, dietary restriction of sodium or increased intake of fruits and vegetables; 2) eliciting barriers and concerns about adoption of each lifestyle modification; 3) summarizing the ‘pros’ and ‘cons’, thereby eliciting positive self-motivational statements about the behavior; 4) providing a menu of options; 5) assessing the participant’s values and goals in connection with his current health behavior pattern; and finally, 6) each encounter will end with a global summary of what was discussed and clarification of an agreed upon action plan.

**Table 2:** Intervention components

	**Motivational interviewing**	**Patient navigation**
Initial Session (in person when possible)	Review lifestyle recommendations in the NHLBI Pamphlets.	Review information in American Cancer Society and Prevent Cancer Foundation DVD/pamphlets:
	• Weight loss for those with a BMI >25	• Colorectal cancer and statistics relevant to black men
	• At least 180 min/week of physical activity	• Risk factors, symptoms and healthy behaviors
	• No more than 1500 mg/day of dietary sodium	• CRC screening options
	• 9-12 daily servings of fruits and vegetables	Explore participant’s barriers (ie lack of knowledge, beliefs and attitudes, fear, logistics) and commitment to CRC screening.
	• 2-3 servings of low fat dairy product	
	• Intake of total fat/saturated fat of < 25% and 75% of total calories respectively	Plan follow-up sessions, colonoscopy referral and/or FIT mailing.
	• Smoking cessation	
	• Medication adherence	
	Barriers to the adoption of TLC are explored and initial goal set.	
Follow-Up/Maintenance Phase (via phone, 30 minutes per session)	1) Assess participant’s motivation and confidence in engaging in given lifestyle behavior	Utilize CEEP (Clarify-Empathize-Explain-Plan) counseling during weekly sessions to assist participants in:
	2) Elicit barriers and concerns about adoption of each lifestyle modification	• Overcoming barriers to CRC screening
	3) Summarize in a non-threatening manner the ‘pros’ and ‘cons’ of participant’s concerns, thereby eliciting positive self-motivational statements about the behavior	• Obtaining colonoscopy referrals or FIT card
	4) Provide a menu of options to the participant based on the nature of barriers elicited from him	• Scheduling and attending appointments
	5) Assess participant’s values and goals, in order to help him link his current health behavior pattern to his core values and life goals	• Purchasing bowel preparation prescription
	6) Global summary of what was discussed and clarification of an agreed upon action plan	• Completing bowel preparation or FIT testing steps
		• Securing appointment escort
		• Troubleshooting insurance issues
		• Understanding CRC screening results
		• Completing treatment follow-up for positive results

#### Monitoring treatment fidelity for the MINT intervention

Fidelity to the intervention protocol is an important consideration for complex interventions. Using the expanded Lichenstein treatment fidelity model developed by the OBSSR Behavior Change Consortium [48,49], we will ensure fidelity through training and ongoing fidelity monitoring. At the beginning of the study, research assistants will participate in an intensive training that consists of the basic physiology of blood pressure, instruction on proper measurement of blood pressure, a primer on behavioral principles of the intervention, and basic interview skills taught through role playing and didactic teaching. In addition, research assistants will receive two full days of information on the principles of MINT techniques, and practice in the delivery of MINT via role-playing. The interventionists will meet biweekly to discuss the previous week’s sessions, troubleshoot, problem-solve and plan for the following week. Bi-monthly booster sessions will be conducted to minimize drift in MINT counseling skills.

All individual MINT sessions will be digitally audio-taped and used for two purposes: 1) individual counselor development and ongoing training/supervision; and 2) systematic review for treatment fidelity. The relatively immediate review and use of recordings will facilitate the feedback and coaching process to develop and maintain the skills of the trained counselor, an approach that has proven to be effective in previous MINT studies [43]. The audio files will also be archived for systematic sampling of a third of participant’s first session and another randomly selected session for adherence to MINT principles as scored by the Motivational Interviewing Treatment Integrity (MITI) scale [50], a validated instrument with satisfactory reliability, convergent and discriminative validity [51].

#### Patient navigation intervention

This intervention will be patterned after the standard New York City Department of Health and Mental Hygiene (NYC DOHMH) patient navigation (PN) protocol [52], and culturally-targeted to black men. It is widely accepted in the health promotion literature that interventions are more effective when they are culturally appropriate. Thus, the effect of a CRC screening navigation intervention in black men may be improved by enhancing its cultural appropriateness through cultural targeting, defined as the use of “a single intervention approach for a defined subgroup that takes into account characteristics shared by the subgroup’s members” [23]. This can be achieved via several approaches [53] that are particularly relevant to CRC screening navigation: 1) an evidential approach that focuses on the impact of a given health issue on the target group through data, including incidence, prevalence or mortality rates; 2) a linguistic approach that focuses on using the preferred language of a specific group; and 3) a sociocultural approach that addresses health-related issues in the context of broader social and cultural values and characteristics of a group. Compared to standard or “generic” interventions, culturally targeted interventions are rated more favorably, are perceived as more credible, and result in greater retention of knowledge over time and greater adherence to recommended health behaviors [54-57].

The goals of the intervention will be to 1) educate participants about CRC screening tests; 2) address participants’ concerns about CRC screening; and 3) help participants overcome barriers to screening (Table 2). Participants randomized to this arm will receive at least two planned sessions with trained patient navigators (PN) to assist them with completion of their choice of CRC screening modality. The first session will occur within seven days of enrollment, and will include culturally-targeted education about 1) CRC in African Americans, 2) risk factors and symptoms of CRC, 3) the need, eligibility and timeline for CRC screening, and 4) the different screening modalities. All participants will be given the American Cancer Society Colon Cancer DVD and Prevent Cancer Foundation Colon Cancer pamphlet. According to consensus guidelines, the main goal of CRC screening should be cancer prevention, and thus, structural examinations like colonoscopy are the screening tests of choice when the resources are available to an individual willing to undergo an invasive test. If participants are unwilling to undergo an invasive test or resources are not available, annual screening with high-sensitivity stool-based tests, like the fecal immunochemical test (FIT) card, is an acceptable option for colorectal screening in average risk adults aged 50 years and older [58].

The initial session will primarily address the financial, structural and psychological barriers to CRC screening completion. After assessing the participant’s commitment to CRC screening, the navigator will refer the participant to the appropriate hospital, and/or will review the FIT completion process and mail three-day Hemoccult® ICT cards to those who request them. The navigator will encourage participants to schedule their colonoscopy appointment within one month or to return the completed FIT cards for testing within two weeks. In the case of colonoscopy, the second session will occur one week before the scheduled appointment, during which the navigator will review bowel preparation information and discuss potential barriers to attending the colonoscopy appointment (that is, obtaining the bowel prep or securing an escort, a requirement for discharge at many medical centers). In the case of FIT, the navigator will call participants to confirm receipt of the FIT card and review the testing steps. A third session might be necessary to confirm colonoscopy completion or relay FIT card results. In the case of multiple participant barriers, the navigator will continue to contact the participant on a weekly basis throughout the six-month intervention until the barriers are resolved and CRC screening is completed.

#### Monitoring fidelity for the patient navigation intervention

As with the treatment fidelity outlined above for the lifestyle intervention, we will adopt the Behavior Change Consortium Treatment Implementation model [48,49]. The patient navigation coordinator for the research team (someone with extensive case management experience) and a co-investigator (Dr. Thompson) will conduct intensive navigator training over the course of two days, utilizing the Colonoscopy Patient Navigator Program Orientation Manual training manual from the New York City Department of Health (NYC DOHMH) Program for Cancer Prevention and Control [52]. The main training goals will be: 1) education (colon cancer, statistics, CRC screening) and 2) outlining the role of patient navigators (appointment referrals and reminders).

The NYC DOHMH modules will be supplemented with strategies to culturally target the navigation protocol to black men. This component includes 1) an overview of CRC statistics specific to black men; 2) a review of known barriers to CRC screening in black adults; 3) application of the CEEP barrier counseling framework (Clarify-Empathize-Explain-Plan) used in previous work [59]; 4) interactive exercises to increase proficiency; and 5) a review of intervention protocol and data collection procedures. Each navigator will also participate in a minimum of three individual, one-on-one skill-building sessions to increase their confidence and mastery; three hours of role plays to simulate realistic PN experiences; a minimum of two hours’ training on intervention documentation and database usage; and at least three hours of one-on-one follow-up training with the patient navigator coordinator. Finally, patient navigators will undertake 56 hours of formal community health worker training organized by the Community Health Worker Network of New York City.

All navigation sessions will be digitally audio-recorded and used for two related purposes: 1) ongoing training/supervision of navigators; and 2) review for treatment fidelity. Ten percent of recorded navigation sessions will be randomly selected and reviewed by the PN expert using a PN fidelity checklist adapted from the Patient Navigator Performance Checklist [60] and informed by the Technology Model of intervention integrity monitoring [61] If this review reveals drift in the intervention delivery protocol by navigators, individual training sessions will be conducted to review relevant areas of training and refine navigator skills through role-play and feedback.

### Outcome assessments

The primary outcomes will be 1) within-patient change in systolic and diastolic BP from baseline to six months and 2) CRC screening rates as determined by colonoscopy report from the primary care provider at six months. For a complete list of measures, please refer to Table 1.

### Primary outcomes

#### Blood pressure

At eligibility screening and six-month follow-up visits, three BP measurements will be taken by a trained RA using an automated BP monitor (Vital Signs Monitor 300 Series; Welch Allyn) following AHA guidelines [62]. The average of the three readings will be used as the measurement for each visit. Uncontrolled BP as defined by the JNC-7 criteria is SBP ≥135 mm Hg or DBP ≥85 mm Hg, with lower thresholds of SBP ≥130 or DBP ≥80 applied to those with co-morbid diabetes or kidney disease [15].

#### CRC screening

CRC screening will be measured primarily by self-report. At six-month follow-up, participants will be asked whether they had a colonoscopy or any other screening for colorectal cancer during the course of the study. As the study will be based in the community and not directly related to any specific CRC screening program, participants who report having had CRC screening and records of it will be requested from the health center. Participants will be asked to sign a medical release form at the time of informed consent to grant permission to the research team to obtain CRC screening reports.

### Secondary outcomes

Fruit and vegetable intake will be measured at baseline and six-month follow-up using a two-item questionnaire developed and validated by Resnicow and colleagues [44]. The items ask how many servings of fruits and vegetables participants eat on an average day.

Physical activity will be measured using the short version of the International Physical Activity Questionnaire (IPAQ-S). The IPAQ-S is a self-report measure that assesses moderate- and vigorous-physical activity, walking, and time spent sitting over a seven-day period. A score is calculated by multiplying the hours and minutes per day spent doing each type of activity by the average metabolic equivalent (MET) and adding each category to the total.

Height, weight and waist circumference will be measured using a stadiometer, with a validated scale (HD-351 Digital Weight Scale; Tanita Corporation of America, Inc.; Arlington Heights, IL, USA), and using a cloth measuring tape, respectively, after the participant has taken off heavy outer garments. All measurements will be recorded to the nearest cm and kg. Height and weight will be used to calculate the body mass index (BMI) for each participant. The difference in weight and waist circumference from baseline to six-month follow-up will be used as a measure of weight-loss over the course of the study. Height will only be measured at baseline.

### Analysis

#### Sample size and power calculations

The sample size is based on the number of participants needed to provide adequate power to test the primary hypothesis, that is, to detect the hypothesized group differences in the primary outcomes. Based on our own clinical experience and estimates from other clinical trials in uncontrolled hypertensive patients [4-6,11,63] we estimate that the cross-sectional standard deviations (SD) of SBP and DBP at baseline will be about 15/12 mm Hg. Conservatively, assuming (a) that the SD at follow-up will be 25% larger in the MINT group, (b) that the correlation between baseline and follow-up BPs will be 0.6 in the CRC patient navigation group and 0.4 in the MINT group (both due to heterogeneity of treatment response), and (c) that there are no barbershop effects (as this is an individual level intervention) on the change in BP, 200 participants per group will provide 86% power to detect a 5 mmHg differential change in SBP and the same power to detect a 4 mmHg differential change in DBP, based on testing the Time X Group interaction in a MANOVA, allowing for heterogeneous variances and serial correlations [64]. Regarding the CRC navigation arm, a sample size of 127 participants per group is needed to provide 90% power to detect a group difference in the rate of completed CRC screening at six months from a baseline rate of 35% [26,27,32,65] to a desired level of 60%. Consequently, we will recruit 240 participants per intervention group (allowing for 20% attrition), which will provide sufficient power to detect the hypothesized difference in both arms of the study. Power analyses were conducted using the R statistical language [64,66].

### Analysis of the primary aims

#### Blood pressure

MANOVA will be used to test the hypothesis that those assigned to MINT counseling will, on average, exhibit greater six-month decreases in SBP and DBP than those assigned to the CRC patient navigation condition. This analysis will have one within-person factor - Time (baseline vs. six-month follow-up) - and one primary between-persons factor (Treatment Group). The outcome measure in this analysis will comprise a linear combination of systolic and diastolic BP.

#### Colon cancer screening

Logistic-type regression will be used to test the hypothesis that those assigned to patient navigation intervention will have significantly higher CRC screening rates compared to those randomized to the lifestyle intervention at six months. Clustered sampling effects due to site and physician will be incorporated using a multilevel modeling approach.

## Discussion

### Public health significance and implications for future research

This protocol will be a unique opportunity to test two interventions simultaneously, offering at least one intervention to each study participant regardless of randomized assignment, eliminating the need for a traditional control group and accompanying ethical issues. Our study will be one of the first to test patient navigation for CRC screening in a community-based setting, thus having the potential to reach individuals without access to regular primary care. These individuals, who are underserved by the healthcare system, are in greater need for preventive care than those with a regular source of primary care and are generally overlooked in clinic-based studies.

Future research should test other innovative methods of addressing health disparities among true community-based populations. Furthermore, our study addresses two health issues among urban black men, yet more research is needed to determine whether there are important differences produced by ethnicity or socioeconomic status within this diverse demographic which may deserve further attention. Finally, it is important to maintain effective programs past the length of a randomized control trial, thereby maintaining the trust of communities and continuing to address health disparities beyond the scope of individual research studies. Research into the efficacy of developing sustainable methods to address health disparities in black communities is essential.

## Trial status

The Mister B trial began recruitment in December of 2009. To date, we have recruited a total of 444 participants from 103 barbershops and other community organizations. Of those, 219 are randomized to receive the MINT intervention, 225 are randomized to receive the PN intervention. Participant recruitment will conclude in approximately four months, and participants will be followed for six months.

## Abbreviations

BMI: Body mass index; BP: Blood pressure; CEEP: Clarify-Empathize-Explain-Plan; CRC: Colorectal cancer; CT: Computed tomography; DBP: Diastolic blood pressure; FIT: Fecal Immunochemical Test; FOBT: Fecal Occult Blood Test; HTN: Hypertension; IPAQ-S: International Physical Activity Questionnaire – short version; IRB: Institutional review board; JNC-7: Seventh Report of the Joint National Committee on Prevention, Detection, Evaluation, and Treatment of High Blood Pressure; MET: Average metabolic equivalent; MINT: Motivational interviewing (lifestyle intervention); MISTER B: *M*ulti-Intervention Study to *I*mprove CRC *S*creening and *t*o *E*nhance *R*isk Reduction in *B*lack Men; MITI: Motivational Interviewing Treatment Integrity; NYC DOHMH: New York City Department of Health and Mental Hygiene; PN: Patient navigation; RA: Research assistant; SBP: Systolic blood pressure; SD: Standard deviation; TLC: Therapeutic lifestyle changes.

## Competing interests

The authors declare that they have no competing interests.

## Authors’ contributions

JR serves as principal investigator for the study at NYU, designed the study and drafted the manuscript. HT designed the patient navigation intervention, helped to design the study and helped to draft the manuscript. HC worked to coordinate the study, refine the implementation of the study and helped to draft the manuscript. JP helped to refine the implementation of the study, helped to refine the motivational interviewing intervention and helped to draft the manuscript, GC helped to refine the patient navigation intervention, refine the study design and implementation and helped to edit the manuscript. LA helped to refine the study implementation, helped to refine the motivational interviewing intervention and helped to edit the manuscript. CBF participated in designing the study and edited the manuscript. MW provided plans for the statistical analysis for the study, power calculations and randomization for the study. MS assisted with study design and refining study implementation and helped to edit the manuscript. GO conceived of and designed the study and helped to edit the manuscript. All authors read and approved the final manuscript.
